# Chemistry and lung toxicity of particulate matter emitted from firearms

**DOI:** 10.1038/s41598-022-24856-5

**Published:** 2022-12-01

**Authors:** Yong Ho Kim, Samuel A. Vance, Johanna Aurell, Amara L. Holder, Joseph Patrick Pancras, Brian Gullett, Stephen H. Gavett, Kevin L. McNesby, M. Ian Gilmour

**Affiliations:** 1 Public Health and Integrated Toxicology Division, Center for Public Health and Environmental Assessment, U.S. Environmental Protection Agency, Research Triangle Park, NC 27711 USA; 2 Center for Environmental Medicine, Asthma and Lung Biology, University of North Carolina, Chapel Hill, NC 27599 USA; 3 Oak Ridge Institute for Science and Education, Research Triangle Park, NC 27711 USA; 4 University of Dayton Research Institute, Dayton, OH 45469 USA; 5 Air Methods and Characterization Division, Center for Environmental Measurements and Modeling, U.S. Environmental Protection Agency, Research Triangle Park, NC 27711 USA; 6 U.S. Army Research Laboratory, Adelphi, MD 20783 USA

**Keywords:** Chemical biology, Physiology, Environmental sciences, Health occupations, Chemistry

## Abstract

Smoke emissions produced by firearms contain hazardous chemicals, but little is known if their properties change depending on firearm and ammunition type and whether such changes affect toxicity outcomes. Pulmonary toxicity was assessed in mice exposed by oropharyngeal aspiration to six different types of smoke-related particulate matter (PM) samples; (1) handgun PM, (2) rifle PM, (3) copper (Cu) particles (a surrogate for Cu in the rifle PM) with and without the Cu chelator penicillamine, (4) water-soluble components of the rifle PM, (5) soluble components with removal of metal ions, and (6) insoluble components of the rifle PM. Gun firing smoke PM was in the respirable size range but the chemical composition varied with high levels of Pb in the handgun and Cu in the rifle smoke. The handgun PM did not induce appreciable lung toxicity at 4 and 24 h post-exposure while the rifle PM significantly increased lung inflammation and reduced lung function. The same levels of pure Cu particles alone and the soluble components from the rifle fire PM increased neutrophil numbers but did not cause appreciable cellular damage or lung function changes when compared to the negative (saline) control. Penicillamine treated rifle PM or Cu, slightly reduced lung inflammation and injury but did not improve the lung function decrements. Chelation of the soluble metal ions from the rifle fire PM neutralized the lung toxicity while the insoluble components induced the lung toxicity to the same degree as the rifle PM. The results show that different firearm types can generate contrasting chemical spectra in their emissions and that the rifle PM effects were mostly driven by water-insoluble components containing high levels of Cu. These findings provide better knowledge of hazardous substances in gun firing smoke and their potential toxicological profile.

## Introduction

Globally, the total number of firearms was estimated to be approximately 1 billion in 2017 with 85% held by civilians, 13% by militaries, and 2% by law enforcement agencies, and was 14% higher than the previous estimate from 2006^1^. There is also a growing trend in firearm types as handguns and rifles became increasingly dominant from 2008^1^. For example, by 2020, handguns and rifles accounted for 75% of annual production of total firearms in the United States^2^. Given the increasing number of firearms, tens of millions of people are exposed to smoke emissions from gun firing activities at various places every year such as military and recreational firing/training ranges^3–7^. Shooters can inhale the smoke directly from firearms but are also exposed from resuspended gunshot residue on their clothing, hands, and other surfaces^3,8^, however it is unclear whether such exposure can cause adverse health effects. In addition, it is important to know whether different ammunitions or types of firearms (e.g., handgun vs. rifle) produce emissions with contrasting chemical and toxicological properties.

Smoke emissions from firing small arms comprise a mixture of particulate matter (PM) and gases that are produced from the combustion of both the primer and the propellant powder, and from the friction of the bullet along the gun barrel^9,10^. Of the complex components in the smoke, metals are major factors of concern because when inhaled they have the potential for causing acute lung injury, and potentially other longer-term disease processes in both the respiratory tract and other organ systems^3,11,12^. Metallic compounds vary with ammunition types but lead (Pb), copper (Cu), zinc (Zn), barium (Ba), and antimony (Sb) are often enriched in the smoke^8,13^. There is also the potential to form the combustion byproduct cyanide (CN), generated when nitrogen oxide (NO) is heated with carbon (C) in the presence of barium oxide (BaO) when firing^14^.

Pb-based ammunition is being actively replaced with non-Pb alternatives in the firearm manufacturing industry, leading to decreases in Pb exposures^15,16^. However, firing small arms still can increase the risk of exposure to Cu and Zn because they are major components of the full metal jacket (casing) covering most Pb and non-Pb bullets. Higher emissions of Cu and Zn have been reported from modern firearms because their barrel is narrower than older models, which increases the friction between the bullet and the barrel, leading to higher metal content in the emissions^10^. Even though gun firing smoke contains hazardous chemicals, little has been done to evaluate potential toxicity and adverse health outcomes from these exposures with most attention being placed on blood Pb levels of shooters at firing ranges^3^. Several studies have, however provided valuable information on chemical analysis and exposure assessment^3,8,13,17,18^.

Here we collected smoke PM from the firing of two different types of small arms, handgun and rifle from either single shot or 3-round bursts (rifle only). PM samples obtained from filter-based monitoring were analyzed chemically prior to PM extraction and toxicity testing. To determine whether emissions vary with rapid firings or different firearms we evaluated the acute lung toxicity of the smoke PM in mice after oropharyngeal aspiration. Lung toxicity was assessed from physiological (breathing parameters) and biological (pro-inflammatory responses) perspectives. Finally, the role of Cu in the pulmonary toxicity of the rifle fire PM was evaluated through attempts at blocking this material with either penicillamine or through chelation of the soluble metal constituents.

## Results

### Physico-chemical characteristics of the gun firing smoke PM

**Figure 1:**
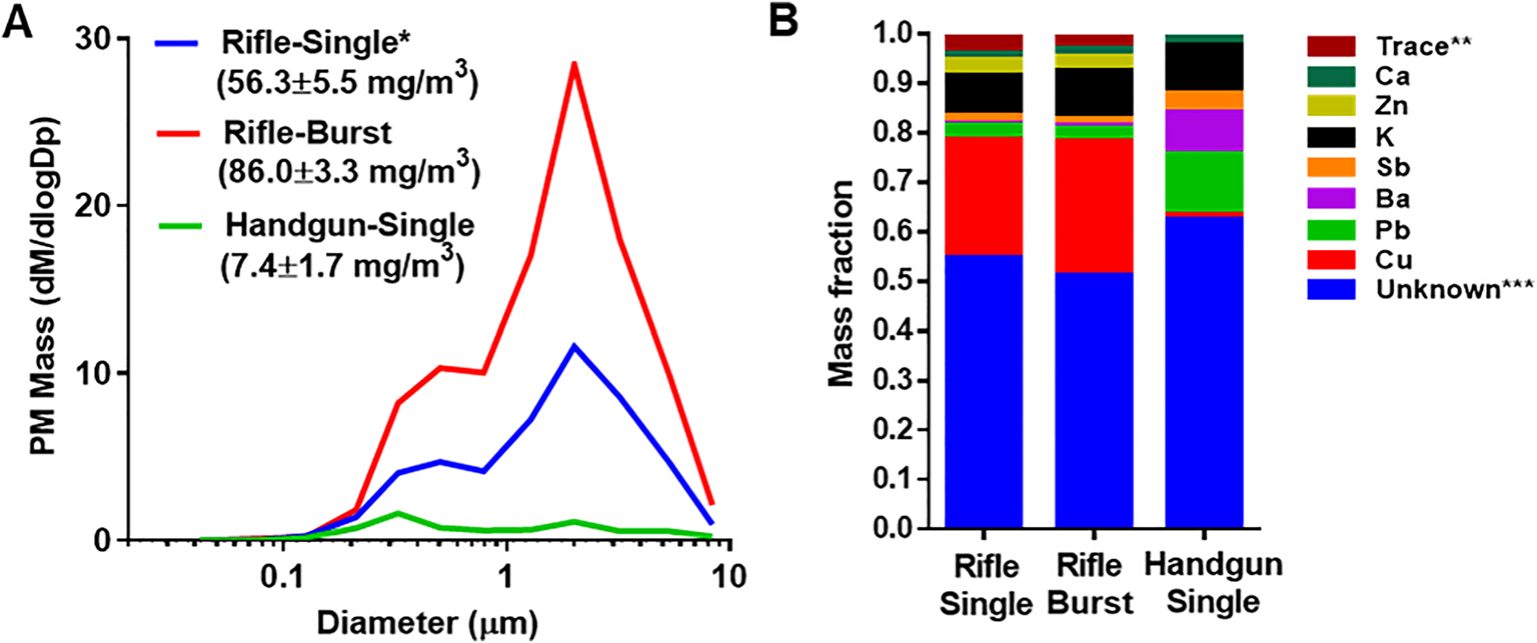
Physico-chemical properties of the rifle and handgun smoke PM. (**A**) PM mass and sizes, (**B**) Inorganic element mass fractions. *Data were collected from the muzzle of the M4 rifle. **Sum of the rest of quantified elements. ***Expected sum of unquantified elements such as N, O, C, and H.

The mass weighted size distributions of the handgun and rifle smoke PM are shown in Fig. 1A. The PM size distribution was similar for the rifle single and burst shots exhibiting a major peak in 2–3 µm range with a second peak in 0.2–0.5 µm, whereas the major peak of the handgun smoke PM was 0.2–0.5 µm diameter followed by the second peak in 2–3 µm, indicating that two particle formation processes, accumulation mode (small particles) associated with combustion processes and coarse mode (large particles) generated by mechanical processes, contributed to the bimodal particle size distributions. The rifle smoke emissions from bursts contained > 10 times higher PM mass than the handgun smoke and were slightly higher (~ 1.5 times) than the rifle smoke from single shots. Inorganic elements accounted for 37% of PM mass in the handgun smoke and 48% and 45% of PM in the rifle smoke from single and burst shots, respectively (Fig. 1B). The highest levels of metals in the handgun and rifle smoke PM were lead (Pb, ~ 12% of PM mass) and copper (Cu, ~ 26% of PM mass), respectively, indicating that chemical components of gun firing smoke PM vary depending on firearm types. Between 52 and 63% of PM mass remained unidentified in the three PM samples (single rifle, burst rifle, and single handgun) were likely to be carbonaceous (organic and elemental carbon) and ionic species (NO_3_^-^ and NH_4_^+^) composed of nitrogen (N), oxygen (O), carbon (C) and hydrogen (H). We further determined solubility of the inorganic elements in the rifle PM. Notably, metals mostly existed as a water-insoluble form (74%, 97%, and 96% of Cu, Pb, and Zn, respectively) and only 21% of the metals (mostly Cu) were found as soluble compounds (Fig. S1). A metal chelator (Chelex) effectively removed Pb and Zn but not Cu from the soluble inorganic compounds, resulting in the low metal PM sample that still contained 10% of Cu in the rifle PM (Fig. S1). Potassium (K) was the second highest contributor to inorganic elements in the gun firing smoke PM presumably from the potassium nitrate (KNO_3_) in the propellant (accounting for 8–10% of PM mass), resulting in an increase in pH of the PM samples (pH 8.35, 8.43, and 8.78 of the handgun, the rifle single, and the burst, respectively) after they were mixed with saline for toxicity testing.

### Lung toxicity and physiology of the gun firing smoke PM

#### Roles of firearm types (handgun vs. rifle)

**Figure 2:**
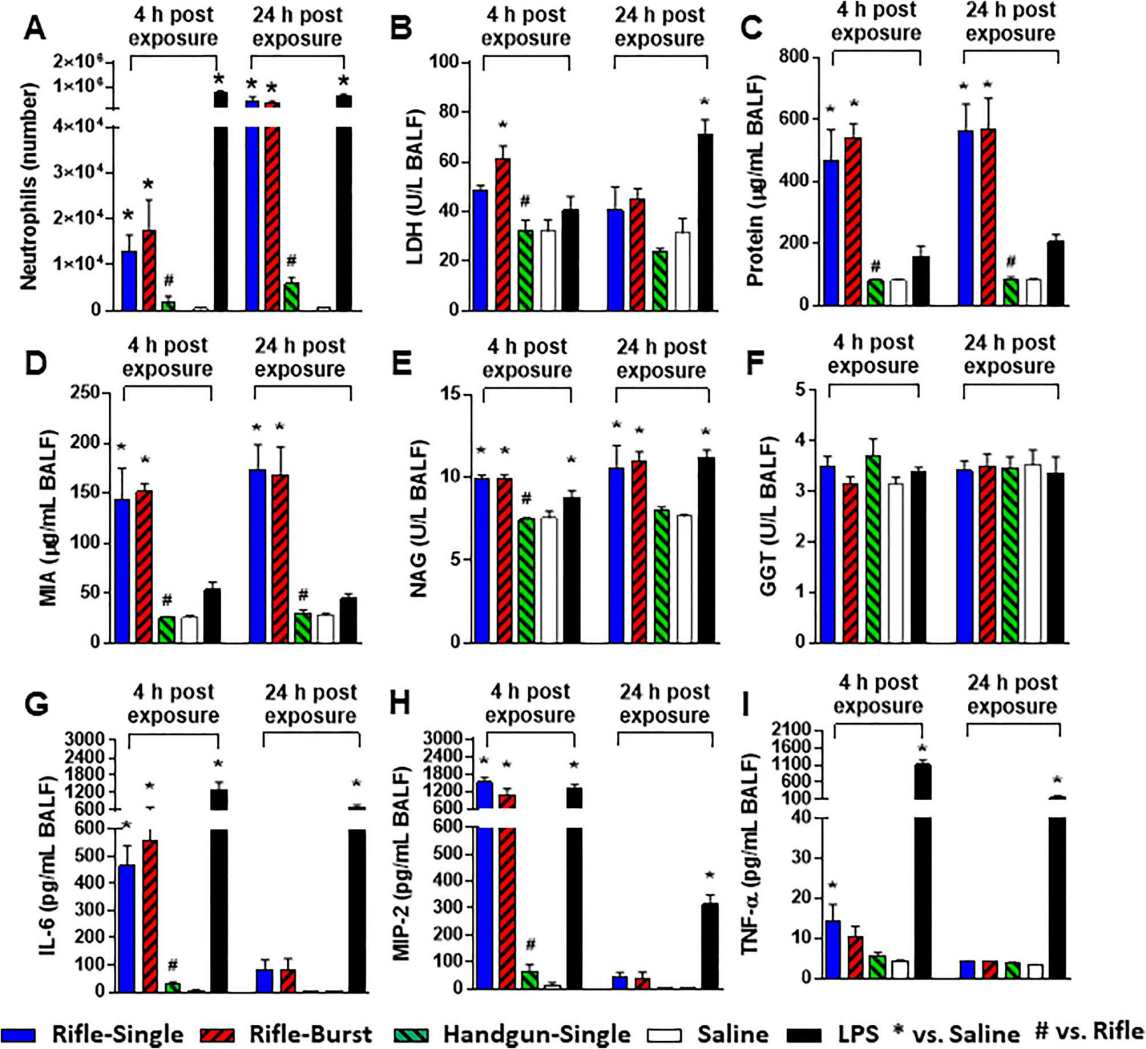
Lung toxicity of mice exposed to the rifle and handgun smoke PM. (**A**) Neutrophil numbers, (**B**) LDH levels, (**C**) Protein levels, (**D**) Micro albumin levels, (**E**) NAG levels, (**F**) GGT levels, (**G**) IL-6 levels, (**H**) MIP-2 levels, (**I**) TNF-α levels. Mice were exposed to the PM (20 µg) by oropharyngeal aspiration and BALF was obtained at 4 and 24 h post exposure. Data are mean ± SEM obtained from 6 mice for each group. *p < 0.05 compared with saline-exposed group (a negative control) from the same time point (one-way ANOVA with post-hoc Dunnett's test). ^#^*p* < 0.05 compared with rifle-exposed group (single or burst) from the same time point (one-way ANOVA with post-hoc Tukey’s test). Mice exposed to 2 µg of lipopolysaccharide (LPS) served as a positive control.

At 4 and 24 h after exposure to an equal mass of the rifle and handgun smoke PM (20 µg of PM), we examined neutrophil accumulation in the lungs and analyzed the bronchoalveolar lavage fluid (BALF) for markers of cellular injury (lactate dehydrogenase (LDH), protein, albumin, N-acetyl-β-D-glucoaminidase (NAG), and γ-glutamyl transferase (GGT)), and pro-inflammatory cytokines (interleukin-6 (IL-6), macrophage inhibitory protein-2 (MIP-2), and tumor necrosis factor-α (TNF-α)) (Fig. 2). The rifle smoke PM (from both single and burst shots) significantly increased neutrophil numbers at both time points whereas the handgun smoke PM had no significant effect (Fig. 2A). A similar pattern of biomarkers of cellular injury (except GGT) was observed from the rifle and handgun smoke PM with greatly increased protein and microalbumin (MIA) levels compared to LDH and NAG (Fig. 2B–F). We also found that the concentrations of IL-6 and MIP-2 were significantly elevated in mice exposed only to the rifle PM (from both single and burst shots) at 4 h, compared with saline controls at the same time point (Fig. 2G and H). Although TNF-α levels were increased with the rifle PM at 4 h, the value was only significantly different from the saline controls for the single shot exposure (Fig. 2I). Some of the hematological parameters (hemoglobin (HB), hematocrit (HCT) and lymphocytes (LY)) were altered by the rifle PM exposure at 24 h but most of them remained unchanged (Table S1).

**Figure 3:**
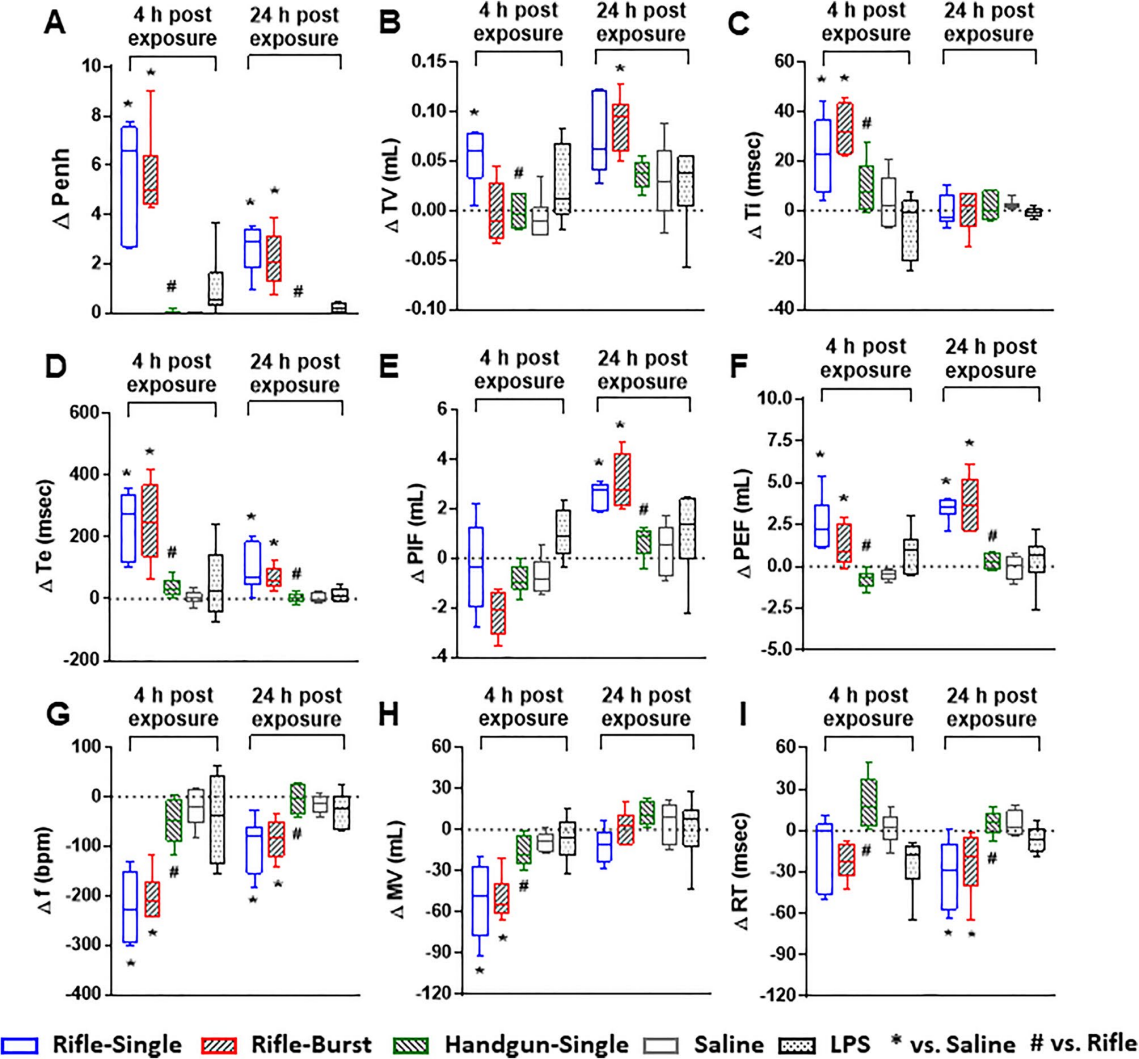
Lung function of mice exposed to the rifle and handgun smoke PM. (**A**) Penh, (**B**) Tidal volume (TV), (**C**) Inspiratory time (Ti), (**D**) Expiratory time (Te), (**E**) Peak inspiratory flow (PIF), (**F**) Peak expiratory flow (PEF), (**G**) Breathing frequency (f), (**H**) Minute ventilation (MV), (**I**) Relaxation time (RT). Mice were exposed to the PM (20 µg) by oropharyngeal aspiration and BALF was obtained at 4 and 24 h post exposure. Data are mean ± SEM obtained from 6 mice for each group. *p < 0.05 compared with pre-exposed (baseline) group from the same time point (one-way ANOVA with post-hoc Dunnett's test). ^#^*p* < 0.05 compared with rifle-exposed group (single or burst) from the same time point (one-way ANOVA with post-hoc Tukey’s test). Mice exposed to 2 µg of lipopolysaccharide (LPS) served as a positive control.

At 4 and 24 h post-exposure, we monitored changes in respiratory parameters in mice (Fig. 3). A significant increase in ventilatory timing (as measured by Penh), indicating potential airflow obstruction, was observed in mice exposed only to the rifle smoke PM (from both single and burst shots) at 4 and 24 h (Fig. 3A). Similarly, the rifle PM exposure significantly elevated tidal volume (TV), inspiratory time (Ti), expiratory time (Te), peak inspiratory flow (PIF), and peak expiratory flow (PEF) at either 4 or 24 h post-exposure (Fig. 3B–F). Other parameters (frequency (f), minute ventilation (MV) and relaxation time (RT)) were significantly decreased in mice exposed to the rifle PM at either 4 or 24 h post-exposure (Fig. 3G–I). The respiratory parameters indicate that lung function changes in mice exposed to the rifle PM were mostly driven by increased expiratory ventilation parameters (e.g., Te and PEF) associated with possible airway narrowing and inflammation. No handgun smoke PM exposed mice had any significant changes in any of the respiratory parameters at either time point.

#### *Roles of Cu content (Rifle PM vs. Cu* + */− Penicillamine)*

**Figure 4:**
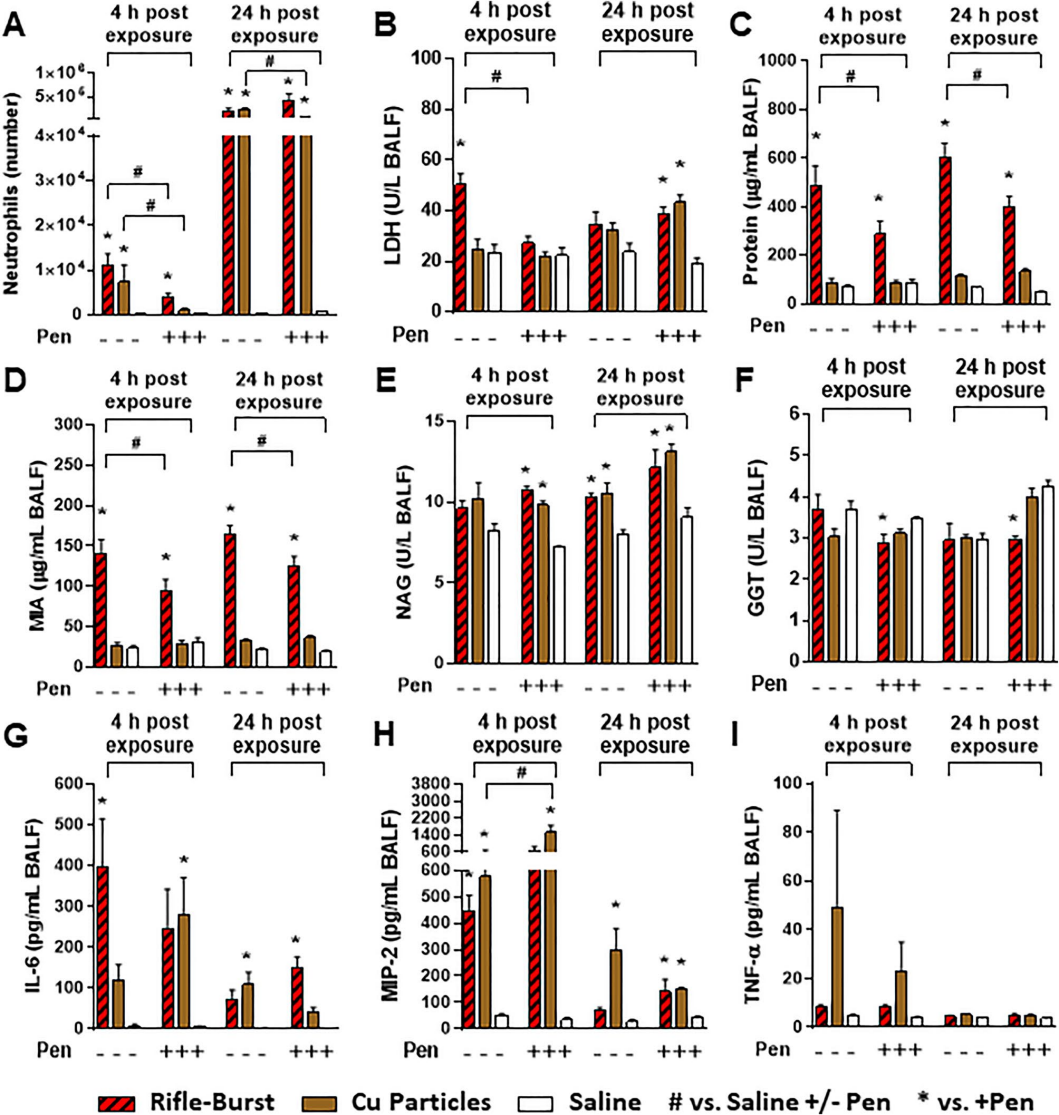
Lung toxicity of mice exposed to the rifle smoke PM and Cu particles with and without Cu chelation (Pen: Penicillamine). (**A**) Neutrophil numbers, (**B**) LDH levels, (**C**) Protein levels, (**D**) Micro albumin levels, (**E**) NAG levels, (**F**) GGT levels, (**G**) IL-6 levels, (**H**) MIP-2 levels, (**I**) TNF-α levels. Mice were exposed to the PM (20 µg) and Cu particles (6 µg) by oropharyngeal aspiration and BALF was obtained at 4 and 24 h post exposure. Data are mean ± SEM obtained from 6 mice for each group. *p < 0.05 compared with saline- or saline + Pen-exposed group (a negative control) from the same time point (one-way ANOVA with post-hoc Dunnett's test). ^#^*p* < 0.05 compared with Pen-exposed group from the same time point (unpaired *t*-test).

Because the handgun smoke PM did not alter any of the lung toxicological parameters studied, we only focused on the rifle smoke PM from bursts for more targeted toxicological assessment. In addition, we attempted to block the effects by concurrent treatment of the rifle fire or free Cu particles (a surrogate for Cu in the rifle PM) with a Cu chelator (Pen; penicillamine). At 4 and 24 h post-exposure, Cu particles significantly increased neutrophil numbers in BALF to the same degree as the rifle smoke PM. The increased numbers of neutrophils associated with the Cu and rifle smoke PM exposure were decreased by Cu chelation but were still significantly higher than saline controls (Fig. 4A). Although the neutrophil number rose under exposure to the Cu particles, LDH, protein, MIA, and GGT levels did not change at 4 and 24 h post-exposure (Fig. 4B–F). Only NAG was increased with exposure to the Cu particles (Fig. 4E). Cu chelation demonstrated some degree of protection against cellular injury with the rifle PM (i.e., LDH, protein, and MIA) but their responses were still significantly higher than saline controls (Fig. 4B–D). Similar to the rifle PM exposure, pro-inflammatory cytokine levels, IL-6 and MIP-2, were significantly increased in mice exposed to the Cu particles (Fig. 4G and H). TNF-α was increased but not significantly different from saline controls (Fig. 4I). None of the cytokine responses was significantly reduced by Cu chelation at any time points. The rifle PM and the Cu particles with and without the chelator changed some hematological parameters (platelet (PLT), plateletcrit (PCT), neutrophil (NE), and lymphocytes (LY)) studied (Table S1).

**Figure 5:**
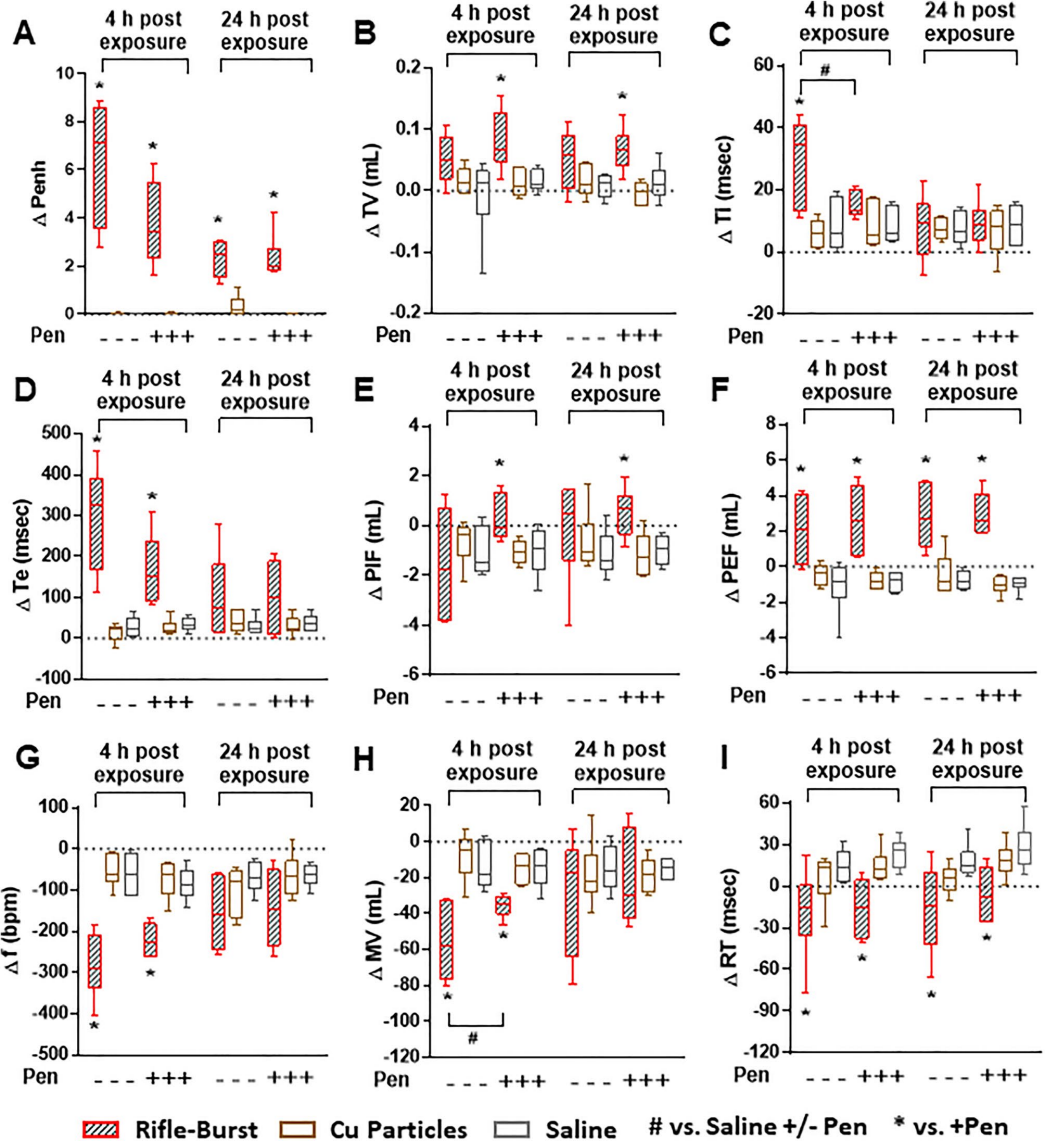
Lung function of mice exposed to the rifle smoke PM and Cu particles with and without Cu chelation (Pen: Penicillamine). (**A**) Penh, (**B**) Tidal volume (TV), (**C**) Inspiratory time (Ti), (**D**) Expiratory time (Te), (**E**) Peak inspiratory flow (PIF), (**F**) Peak expiratory flow (PEF), (**G**) Breathing frequency (f), (**H**) Minute ventilation (MV), (**I**) Relaxation time (RT). Mice were exposed to the PM (20 µg) and Cu particles (6 µg) by oropharyngeal aspiration and BALF was obtained at 4 and 24 h post exposure. Data are mean ± SEM obtained from 6 mice for each group. *p < 0.05 compared with pre-exposed (baseline) group (saline- or saline + Pen-exposed group) from the same time point (one-way ANOVA with post-hoc Dunnett's test). ^#^*p* < 0.05 compared with Pen-exposed group from the same time point (unpaired *t*-test).

At 4 and 24 h post-exposure, we found that the Cu particles did not alter any of the respiratory parameters (Fig. 5A–I). Significant increases in Penh, TV, Te, PIF, and PEF levels observed in mice exposed to the rifle smoke PM were largely unchanged with Cu chelation at 4 and 24 h post-exposure, with the exception of a decreased in Ti (Fig. 5A–F). Similarly, reduced respiratory parameters (i.e., f, MV and RT) for the rifle PM did not fully recover to baseline with attempts to chelate the Cu at any time points, except MV was improved (Fig. 5G–I).

#### *Roles of water-insoluble components (soluble vs. insoluble* + */− Chelex)*

**Figure 6:**
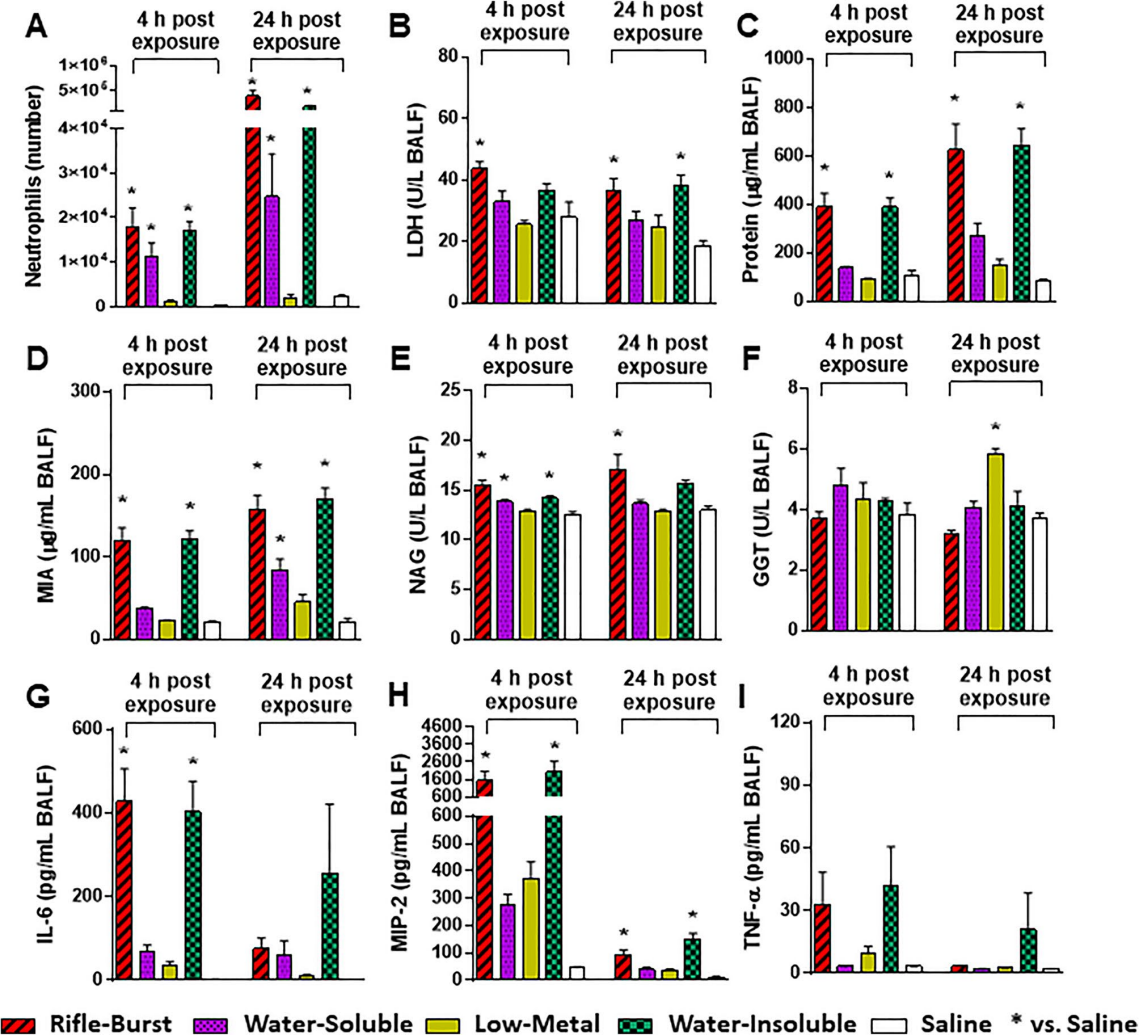
Lung toxicity of mice exposed to the rifle smoke PM, water-soluble components, water-soluble components without metal ions (low-metal), and water-insoluble components. (**A**) Neutrophil numbers, (**B**) LDH levels, (**C**) Protein levels, (**D**) Micro albumin levels, (**E**) NAG levels, (**F**) GGT levels, (**G**) IL-6 levels, (**H**) MIP-2 levels, (**I**) TNF-α levels. Mice were exposed to the PM (20 µg) by oropharyngeal aspiration and BALF was obtained at 4 and 24 h post exposure. Data are mean ± SEM obtained from 6 mice for each group. *p < 0.05 compared with saline-exposed group (a negative control) from the same time point (one-way ANOVA with post-hoc Dunnett's test).

At 4 h post-exposure, the soluble and insoluble samples significantly increased neutrophil numbers in BALF, but only the insoluble sample continued to increase the neutrophil number to a similar degree as the rifle smoke PM at 24 h post-exposure (Fig. 6A). Neutrophil numbers were not significantly increased after exposure to the low-metal sample (water-soluble components with removal of metal ions) at either time point. We also found that the soluble and low-metal samples caused few significant increases in LDH, protein, and MIA levels (except the MIA level of the soluble at 24 h), while significant increases in cellular injury were observed in mice exposed to the insoluble sample at 4 and 24 h post-exposure (Fig. 6B–D). Small (but significant) increases in NAG and GGT were observed for the soluble and insoluble (NAG at 4 h) samples and the low-metal samples (GGT at 24 h) (Fig. 6E and F). Pro-inflammatory cytokine responses in BALF were clearly associated with the insoluble components (Fig. 6G–I). The concentrations of IL-6 and MIP-2 were significantly elevated at 4 h after exposure to the insoluble sample and their responses were similar to (not statistically different from) the ones induced by the rifle smoke PM. TNF-α levels were also increased for the insoluble sample at 4 h but not significantly different from saline controls. None of the exposures to these three different components of the rifle PM significantly altered any of the hematological parameters studied at any time points (Table S1).

**Figure 7:**
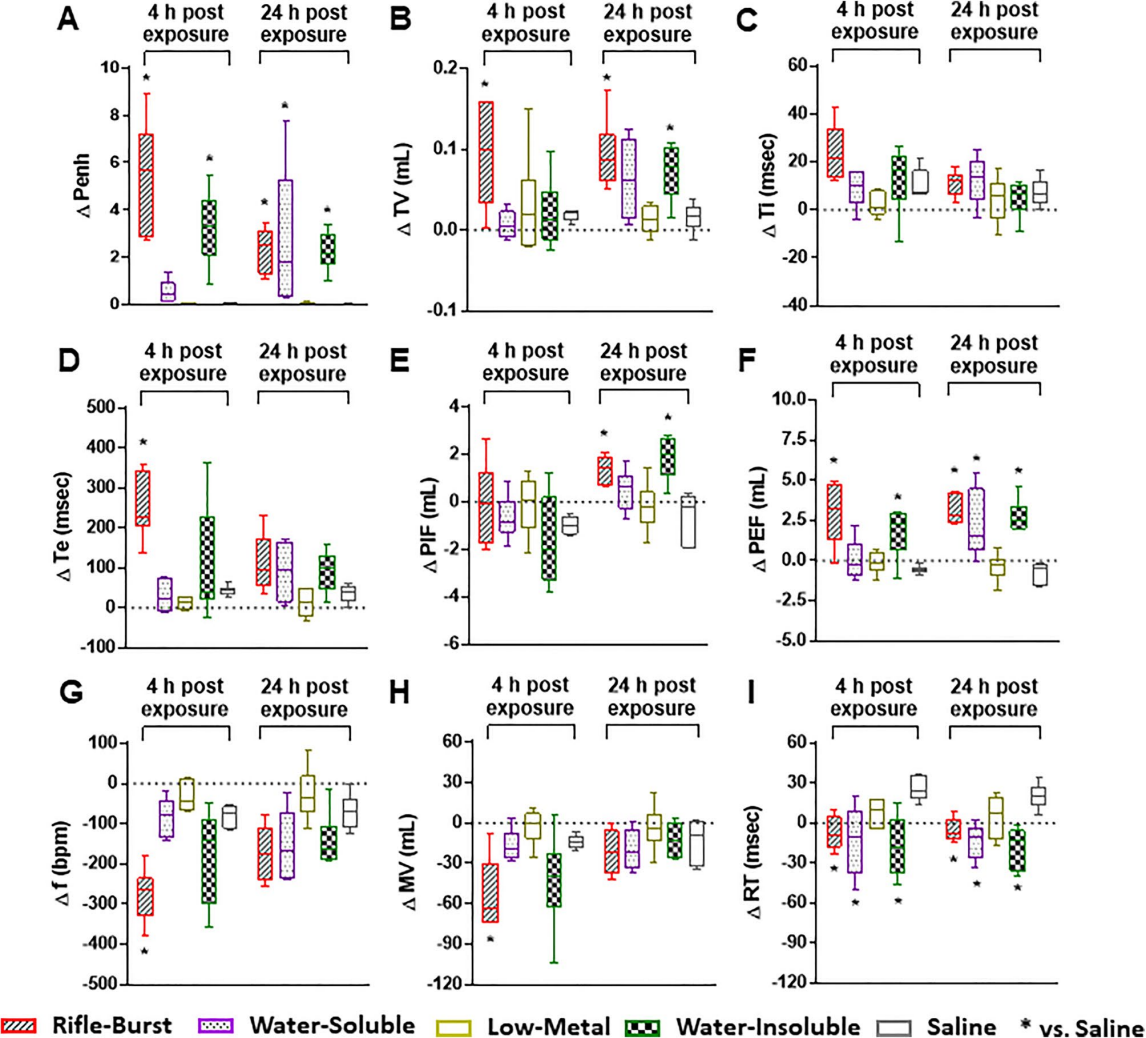
Lung function of mice exposed to the rifle smoke PM, water-soluble components, water-soluble components without metal ions (low-metal), and water-insoluble components. (**A**) Penh, (**B**) Tidal volume (TV), (**C**) Inspiratory time (Ti), (**D**) Expiratory time (Te), (**E**) Peak inspiratory flow (PIF), (**F**) Peak expiratory flow (PEF), (**G**) Breathing frequency (f), (**H**) Minute ventilation (MV), (**I**) Relaxation time (RT). Mice were exposed to the PM (20 µg) by oropharyngeal aspiration and BALF was obtained at 4 and 24 h post exposure. Data are mean ± SEM obtained from 6 mice for each group. *p < 0.05 compared with the pre-exposed (baseline) group from the same time point (one-way ANOVA with post-hoc Dunnett's test).

At 4 and 24 h post-exposure, only the water-insoluble sample from the rifle fire PM significantly induced respiratory changes to the same degree as the rifle smoke PM (Fig. 7). Penh, TV, PIF and PEF were significantly increased in response to the insoluble sample at 4 or 24 h post-exposure (Fig. 7A–F). Ti and Te levels in association with the insoluble sample were also increased but not significantly different from baseline at any time points. Similarly, the insoluble sample decreased f, MV, and RT levels at 4 or 24 h post-exposure but significant decreases were only observed in RT (Fig. 7G–I). The water-soluble sample induced some changes in respiratory parameters (Penh, PEF, and RT) that were lesser in magnitude than the changes caused by the rifle PM; these responses were markedly reduced or neutralized with chelation (the low-metal sample) at all time points.

## Discussion

### Not all gun firing smoke is created equal

Most ammunition consists of the same basic components including bullets, primers, powder and cases but their formulations vary by bullet and different types of firearms. When ammunition is fired, it emits gases and particles with different physico-chemical properties depending on the chemical composition of the ammunition and how the ammunition is discharged from different firearms. We demonstrated that handgun and rifle ammunition when fired emitted largely different chemical species of PM in the smoke (high levels of Pb in handgun smoke, whereas rifle smoke had high levels of Cu) (Fig. 1). Given that the chemical components (e.g., primers and powder) of the ammunitions themselves and the nature of the combustion reactions within the barrel are very similar, our findings suggest that the mechanical action (e.g., friction and velocity) of the bullet on the barrel is an important factor that drives different firing smoke emissions^13^. This is consistent with data published previously showing high concentrations of Cu and Zn in the rifle smoke, but Pb in the handgun smoke even from firing unleaded ammunition^19^. In a similar vein, other studies report that the friction of the bullet in the barrel contributes to gun firing smoke emission characteristics^10,12^. For example, in a comparison of harder steel core ammunition, which results in larger abrasion of the bullet and more metal fragments in the smoke, to Pb core ammunition, the steel core produced more Cu and Zn than the Pb core ammunition when fired in the same rifle. This suggests that exposure to metals from firing ammunition is more affected by barrel design than bullet type^20^. It has been reported that smoke particle sizes (a major peak at 1–4 µm) were largely unchanged from firing different ammunitions^10,18,19^. Our data also showed that the largest mass fraction of PM in the handgun and the rifle smoke was in the size range 1–3 µm (Fig. 1). These particle sizes are mostly associated with metal particle formation from bullet abrasion^10^. Notably, we observed the main peak with a shoulder of smaller particles (< 0.3 µm). These particles (mostly carbonaceous particles) are more likely to be produced as a result of the combustion of the primer and propellant in the bullet, creating a bimodal particle size distribution^8,10,17^. Taken together, our findings suggest that Pb-free rifle ammunitions are a health concern because they produce large amounts of other metals (notably copper) in the respirable particle size range. Also, gun firing smoke cannot be considered as a single hazard and risk assessment should perhaps be conducted separately for each ammunition and firearm type.

### Lung toxicity is associated with Cu and water-insoluble fraction of the rifle PM

We demonstrated that the PM sampled from firing of the rifle but not the handgun ammunition caused biological and physiological damage to the lungs following an acute exposure and the toxicity outcomes were not significantly different between the single and the burst shots (Figs. 2 and 3). Our results are in line with the finding of a previous study demonstrating that gunshot emissions from a rifle induced higher levels of oxidative stress and the pro-inflammatory marker IL-8 in lung epithelial cells than those from a handgun^19^. More importantly, the PM dose (20 µg) of the gun firing smoke in this study was 5 times lower than that of ambient pollutants (100 µg) used in our previous studies^21–23^ but toxicity responses were substantially higher, suggesting that the rifle ammunition emits more toxic chemicals than other combustion sources we previously studied (diesel, biomass smoke, and ambient PM from various locations). Of the complex chemical components in the gun firing smoke, inorganic elements are major factors of concern because they accounted for nearly half of PM mass in this study. Specifically, Cu and Zn are of particular interest not only because they are toxic but also because they were the dominant metals in the rifle smoke PM (~ 30% of PM mass). This is consistent with the previous findings demonstrating that high emissions of Cu and Zn from firing of small firearms are attributed to cytotoxic effects (decreased levels of viability and increased levels of DNA damage) in lung cells^10^ and general health problems (increased levels of neutrophils and inflammatory markers in the blood) in military personnel^11,12^. Similarly, we observed a greater increase in the number of blood neutrophils in mice exposed to the rifle smoke PM. Our findings clearly showed that the rifle PM (characterized by high level of Cu) significantly increased disruption of cell membrane integrity (protein), vascular permeability (MIA), release of cytoplasmic/lysosomal (LDH and NAG) enzymes and pro-inflammatory mediators (IL-6, MIP-2, and TNF-α) in BALF at 4 or 24 h post-exposure (Fig. 2). Similarly, lung function parameters in this study were also significantly increased only after exposure to the rifle PM. Exposure to the rifle PM (either single or burst shot) caused indicators of airflow obstruction in the lungs, including increased tidal volume, fast breathing frequency, short relaxation time, decreased minute ventilation and prolonged inspiration and expiration (Fig. 3). Such breathing patterns are often seen in obstructive lung disease where exhaled air is slowly expelled with faster breathing rates, short relaxation time, and decreased minute ventilation and tidal volume because of damage to the lungs or narrowing of the airways^24,25^. Notably, the increased tidal volume observed in this study is probably because the rifle PM exposure leads to higher ventilation and more oxygen delivered to compensate for the decreased lung volume. Such results are consistent with those from other studies for human health which show shortness of breath, coughing, and decline in lung function after exposure to smoke emissions from firing small arms^12,20^. It is interesting to note that lipopolysaccharide (LPS) exposed mice developed significant inflammation in the lungs but did not change breathing parameters at any time point. This limitation has been discussed in a published paper demonstrating that LPS elicits a significant change in inflammatory responses but does not always cause changes in contraction and relaxation of small airways^26^.

Although we demonstrated that Cu, the primary metal in the rifle smoke PM, has the potential to cause lung injury after exposure, we further investigated the form of Cu responsible for the toxicity outcomes. Since firing ammunition is associated with oxygen-deficient combustion and mechanical friction with high temperature^18^, Cu compounds in the smoke emission are likely more complicated than pure Cu formation. We showed that pure Cu particles, which have a comparable size and mass to Cu in the rifle smoke PM, induced neutrophil influx to the same degree as the rifle PM but did not alter indicators of vascular leakage or membrane disruption in the lungs (no changes in LDH, protein or MIA) over the time studied (Fig. 4). This is consistent with the finding that neutrophil recruitment occurs without any significant changes in epithelial or endothelial permeability^27,28^. This also suggests that complex Cu compounds (e.g., Cu oxides, Cu sulfate, or Cu cyanide), and not pure Cu particles per se played an important role in increasing the permeability of the alveolar/capillary barrier and in producing cellular toxicity during inflammatory responses^29^. Also, a lack of beneficial effects of penicillamine on the toxicity responses suggests that of the complex Cu compounds, water-insoluble complexes (Cu compounds not bound to penicillamine) were major contributors to the acute lung injury and airway obstruction (Figs. 4 and 5). Although penicillamine is known to chelate Cu ions to prevent Cu-mediated toxicity, it is also reported that reactive oxygen species can be produced when penicillamine is incubated with Cu sulfate (CuSO_4_) in a solution, leading to inflammatory responses^30^. Since emissions from firing small arms have high Cu compounds (mostly emitted from the bullet jacket), if potassium cyanide (KCN) can be produced from the reaction of HCN and KNO_3_ in the firing emission, Cu sulfate can further react with KCN to form Cu cyanide (CuCN) which is insoluble and highly toxic and causes severe health effects after exposure^31^. It should be noted that although the pure Cu particles did not change breathing parameters at 24 h after exposure, they induced significant neutrophilia. Thus, we cannot rule out their long-term effects on lung function. In support of this, some studies reported that functional changes in mouse lungs were not significantly altered until 48 h post-exposure although increased neutrophil numbers were observed at early time points (< 24 h post-exposure)^32,33^. Similarly, other studies have suggested that since protein leakage occurs after neutrophil recruitment, the protein content is more predictive of the lung function changes, whereas the degree of neutrophilia is not^34,35^. The handgun smoke PM did not change any of lung toxicity and breathing parameters at any time points in this study. However, since we found large fractions of Pb (12% of PM mass) and Sb (4% of PM mass) in the handgun PM which can increase risks of respiratory and systemic diseases including cancer^36–38^, long-term exposure studies are needed to better understand the association between metals in the hand gun smoke emission and adverse health effects. Numerous studies have shown that shooting at firing ranges results in elevated levels of airborne Pb, Zn, or Cu and a variety of adverse health outcomes, but it is unclear which metals discharged from ammunitions are linked to specific health risks of gun firing smoke exposures^3,39^.

Given that water-insoluble Cu complexes (e.g., Cu oxides or Cu cyanide) showed similar toxicity as whole rifle PM, it is also possible that the toxicity responses could be attributed to other insoluble compounds. Expectedly, the insoluble fraction of the rifle PM could induce the same degree of lung toxicity (biomarkers and lung function) observed with the rifle PM exposure (Figs. 6 and 7). This is not surprising because most of the metals in the rife PM were found to be apparently more insoluble (Fig. S1). The role of insoluble compounds in PM-induced lung toxicity has been extensively studied^40–42^. A study using insoluble extracts of ambient PM samples with a high level of redox-active transition metal content demonstrated that similar degrees of neutrophilic inflammation and protein leakage occurred between the insoluble and total fraction of PM^43^. Of note, another study reported that insoluble organic compounds of PM were largely responsible for extracellular thiol oxidative activity in lung cells after exposure^44^. It is also well recognized that exposures to metal fumes containing insoluble Zn or Cu oxides increase incidence of acute but severe respiratory tract inflammation, fever, and muscle pain^45,46^. Moreover, some studies have indicated that long-term exposure to Cu fume increases risks of mortality for chronic non-malignant diseases of the respiratory system and even lung cancer^47–49^. These adverse effects were also confirmed in many in vivo and in vitro models where exposures to CuO or ZnO particles induced acute inflammatory responses and DNA damage that are associated with intracellular production of reactive oxygen species^50–52^.

While much attention has focused on the role of insoluble compounds, it should be noted that the rifle PM-induced lung toxicity was also mediated, at least in part, by water-soluble compounds (e.g., soluble organics and metal ions) (Figs. 6–7). Interestingly, the significant neutrophil influx triggered by the soluble compounds (i.e., Cu ions) did not change most of the lung injury biomarkers and lung function parameters but showed significantly increased vascular permeability (Fig. 6D) and airway obstruction (Fig. 7A, F and I) at 24 h post-exposure, suggesting the potential for delayed toxic effects. In addition, although Cu is essential for cellular physiology, excess Cu ions can bind with and affect the structure and integrity of DNA^53,54^. Thus, more chronic health consequences from exposure to Cu ions which were the major metal species in the soluble fraction of the rifle PM should not be ignored.

## Conclusions

We demonstrated that the handgun smoke PM caused no lung toxicity at 24 h post-exposure, while the rifle smoke PM at the same concentration showed strong toxicity (including injury, inflammation and decrements in lung function) following a single instillation exposure in association with the high level of Cu (26% of PM mass). Cu particles (as a surrogate for Cu compounds in the rifle PM) alone increased neutrophil numbers but did not alter indicators of lung injury, pro-inflammatory cytokines, and lung function. Also, water-soluble constituents of the rifle PM increased neutrophil numbers and some indicators of lung function and these responses were neutralized when metals were chelated out. All biological and physiological responses (inflammation, injury and lung function changes) tested were reproduced with the water-insoluble fraction of the rifle PM (mostly metals). Overall, our findings can identify the least and /or most toxic components of the gun firing smoke emissions tested and provide mechanistic information on the potential cause. However more work is needed to fully speciate the smoke emissions from the firearms to provide causal links between the chemical constituents and bioactivity and also explore effective interventions that may help reduce exposure to the smoke at firing activities. Finally, knowledge of the relative toxicity of smoke emissions from different ammunitions or types of firearms will inform hazard identification and risk assessment approaches related to the smoke exposures.

## Methods

### Gun firing smoke PM sampling

Gun firings were conducted at the U.S. Army Research Laboratory (Aberdeen Providing Ground, MD). Two firearm types, an M4 rifle using M855A1 ammunition (Pb free copper jacketed steel core) and a 9 mm handgun (copper jacketed Pb slug), were tested in Plexiglas enclosures which allowed rapid sampling of the smoke PM after firing^8^. The propellant and primers from both ammunitions were similar: propellant (nitrocellulose, nitroglycerine, graphite, diphenylamine, ethyl centralite, bismuth, potassium nitrate) and primer (lead styphnate, pentaerythritol tetranitrate, aluminum powder, barium nitrate, antimony sulfide, tetracene). Smoke PM (PM_10_) from single shot (5 firings; total 5 shots) and 3-round bursts (5 firings; total 15 shots) was collected from the muzzle or breech of the M4 rifle (designated rifle smoke PM). Smoke PM was collected from both the muzzle and breech of the handgun during 4 single shot firings (designated handgun smoke PM). The time between each round fired was approximately 20 min for cleaning of test enclosures and resetting of instruments. PM was sampled with SKC impactors on 47 mm Teflon filters with a pore size of 2.0 µm using a Leland Legacy sample pump (SKC Inc., Eighty Four, PA). PM size distributions were measured after the gun firing with an Electrical Low Pressure Impactor (ELPI, Dekati Ltd., Kangasala, Finland). The ELPI measures size resolved particle mass on a series of polycarbonate substrates (10 bins from 28 to 10 µm).

### Gun firing smoke PM samples

The PM samples extracted from filters were suspended in sterile saline at a final concentration of 0.4 mg/ml. The rifle smoke PM (burst shot) was further processed to separate water-soluble components from water-insoluble components. The rifle PM in saline was centrifuged (at 2500 × *g* for 30 min) and the supernatant was used as the water-soluble rifle PM sample (water-soluble sample) and the centrifuged materials were resuspended in saline and used as the water-insoluble rifle PM sample (water-insoluble sample). The supernatant was also mixed with Chelex (metal chelator; Chelex 100, Sigma Aldrich) at 100 mg/ml to remove water-soluble metals^55^. After centrifugation, the chelated supernatant was used as a low metal water-soluble rifle PM sample (low-metal sample). To determine whether metals in the gun fire smoke PM were driving the toxic effects (e.g., high levels of Cu in the rifle smoke), Cu microparticles (5 µm in diameter, 99.8% purity; Sigma Aldrich) were suspended in saline at a concentration of 0.12 mg/ml (similar mass of Cu in the rifle PM). The rifle PM and Cu particles were also mixed with penicillamine (Cu chelator; USP reference standard; Sigma Aldrich) at 0.12 mg/ml to remove Cu ions in the samples. All samples were vortexed and sonicated to ensure homogeneity and then used for toxicity testing and inorganic elemental analysis. The gun firing smoke PM samples were digested in 3:1 aqua regia mixture to leach trace elements and assayed for 46 target elements by high-resolution-magnetic sector field inductively coupled plasma mass spectrometry (HR-ICP-MS; Element 2, Thermo Scientific).

### Experimental animals

Adult pathogen-free female CD-1 mice (~ 28 g body weight) were purchased from Charles River Breeding Laboratories (Raleigh, NC), housed in groups of 3 in polycarbonate cages with hardwood chip bedding at the U.S. EPA Animal Care Facility (accredited by the Association for Assessment and Accreditation of Laboratory Animal Care), and were maintained on a 12-h light-to-dark cycle at 22.3 ± 1.1 °C temperature and 50 ± 10% relative humidity. Mice were weighed and randomly divided into 32 groups of 6 mice each for each exposure condition. Mice were given access to rodent chow and water ad libitum and were acclimated for at least 10 days before the study began. Mice were treated humanely and with regard for alleviation of suffering. This study was approved by the EPA Institutional Animal Care and Use Committee and conducted following the Guide for the Care and Use of Laboratory Animals published by the National Research Council and the recommendations in the ARRIVE guidelines and all the methods were performed in accordance with the relevant guidelines.

### Exposure to the gun firing smoke PM

We administered 20 µg PM samples into the mouse lungs at in 50 µL saline by oropharyngeal aspiration performed in anesthetized mice using vaporized anesthetic isoflurane as described previously^56^. The selection of PM dose (20 µg) was based on extreme exposure levels of PM at indoor firing ranges. If exposures are near or close to firearms (assuming > 60 mg/m^3^ of PM based on the PM measurement in this study), PM deposited in the human lungs for 15 min would be 25.7 ng/cm^2,56^. In the present study, the PM dose (20 µg) to the mouse lung was calculated to be 25 ng/cm^2^, assuming the respiratory deposition fraction by the oropharyngeal aspiration method and surface area of 0.81 and 642 cm^2^, respectively^57,58^, and appeared to be relevant to the inhaled gun firing smoke PM concentrations in human lungs (25.7 ng/cm^2^). We instilled a separate cohort of mice with 2 µg of lipopolysaccharide in 50 µL saline (LPS; *Escherichia coli* endotoxin; 0111:B4 containing 10^6^ unit/mg material, Sigma, St. Louis, MO) as a positive control to demonstrate maximal responsiveness to this well characterized inflammatory agent. We also instilled additional mice with 50 µL saline alone as a negative control.

### Breathing parameter assessment

Mice were tested for breathing parameters before exposures, and after exposure approximately 1 h before euthanasia. Breathing parameters were assessed using a whole-body plethysmography (WBP) system (Emka Technologies, Falls Church, VA) as previously described^59^. Breathing parameters measured include minute ventilation (MV), tidal volume (TV), breathing frequency (F), relaxation time (RT), inspiratory (Ti) and expiratory (Te) time, and peak inspiratory (PIF) and peak expiratory (PEF) flow. In addition, time and flow rate parameters were used to evaluate an index of ventilatory timing (enhanced pause; Penh). In this system the mouse had complete freedom of movement in a small clear plastic chamber (3.5" diameter × 2.5" height).

### Lung toxicity assay

At 4 and 24 h post-exposure, mice were euthanized by overdose with sodium pentobarbital and phenytoin sodium i.p. (Euthasol; Virbac AH Inc., Fort Worth, TX). Blood was collected by cardiac puncture, and hematology values were measured using a Coulter AcT 10 Hematology Analyzer (Beckman Coulter Inc., Miami, FL). Bronchoalveolar lavage fluid (BALF) was collected from the right lung lobes and used to determine the total cell count and differential analysis of macrophage and neutrophil numbers. Total BALF cell count of each mouse was obtained by a Coulter counter (Coulter Co., Miami, FL). Albumin and total protein concentrations in BALF were measured by the SPQ test system (DiaSorin, Stillwater, MN) and the Coomassie plus protein assay (Pierce Chemical, Rockford, IL) with a standard curve prepared with bovine serum albumin (Sigma), respectively. Concentrations of lactate dehydrogenase (LDH) and γ-glutamyl transferase (GGT) in BALF were determined using commercially available kits (LDH-L Reagent and Gamma GT Reagent, Thermo Scientific, Middletown, VA). Activity of N-acetyl-β-D-glucoaminidase (NAG) in BALF was determined using a NAG assay kit (Roche Applied Science, Indianapolis, IN). All biochemical assays were modified for use on the KONELAB 30 clinical chemistry spectrophotometer analyzer (Thermo Clinical Lab Systems, Espoo, Finland) as described previously^56^. Concentrations of tumor necrosis factor-α (TNF-α), interleukin-6 (IL-6), and macrophage inhibitory protein-2 (MIP-2) in BALF were determined using commercial multiplexed fluorescent bead-based immunoassays (Milliplex Map Kit, Millipore Co., Billerica, MA) measured by a Luminex 100 (Luminex Co., Austin, TX) following the manufacturer’s protocol. The limits of detection (LOD) of each cytokine were 6.27, 3.28, and 29.14 pg/mL for TNF-α, IL-6, and MIP-2, respectively. All values below these lowest values were replaced with a fixed value of one-half of the LOD value.

### Statistical analysis

For analysis of lung toxicity (pro-inflammatory cytokines, LDH, NAG, GGT and hematology values) and lung function data, we used one-way analysis of variance (ANOVA) followed by Dunnett’s multiple comparison test to compare the biological and physiological responses to a saline (negative) control. In order to compare the responses across different exposure groups (i.e., handgun vs. rifle smoke PM), we used Tukey’s post hoc test. We also performed an unpaired *t*-test (two-tailed) to determine whether significant differences existed between two exposure groups (with and without penicillamine treatment). This analysis was performed using GraphPad Prism software (version 6.07, GraphPad Software Inc., San Diego, CA). We modeled the lung toxicity potencies (# neutrophils) of the gun firing smoke PM with negative binomial regression in R Statistical Software (version 3.3.2, R Foundation for Statistical Computing, Vienna, Austria)^59^. Data were expressed as mean ± standard error of the mean (SEM). The statistical significance level was assigned at a probability value of *p* < 0.05.

## Supplementary Information

Supplementary Information.

## Data Availability

The data sets used and/or analyzed during the current study are available from the corresponding author on request.
