# Supplementary material for: Chemistry and lung toxicity of particulate matter emitted from firearms
Source: Sci Rep. 2022 Dec 1;12:20722. doi: 10.1038/s41598-022-24856-5 (PMC9715551; doi:10.1038/s41598-022-24856-5)
Supplement: Supplementary file 1 — Supplementary Information. [file 41598_2022_24856_MOESM1_ESM.docx]

**Supporting Information for**

**Chemistry and Lung Toxicity of Particulate Matter Emitted from Firearms**

Yong Ho Kim^1, 2*^, Samuel A. Vance^3^, Johanna Aurell^4^, Amara L. Holder^5^, Joseph Patrick Pancras^1^, Brian Gullett^5^, Stephen H. Gavett^1^, Kevin L. McNesby^6^, M. Ian Gilmour^1*^

^1^Public Health and Integrated Toxicology Division, Center for Public Health and Environmental Assessment, U.S. Environmental Protection Agency, Research Triangle Park, NC 27711, USA

^2^Center for Environmental Medicine, Asthma and Lung Biology, University of North Carolina, Chapel Hill, NC 27599, USA

^3^Oak Ridge Institute for Science and Education, Research Triangle Park, NC 27711, USA

^4^University of Dayton Research Institute, Dayton, OH 45469, USA

^5^Air Methods and Characterization Division, Center for Environmental Measurements and Modeling, U.S. Environmental Protection Agency, Research Triangle Park, NC 27711, USA

^6^U.S. Army Research Laboratory, Adelphi, MD 20783, USA

^*^Correspondence: [kim.yongho@epa.gov](mailto:kim.yongho@epa.gov) and [gilmour.ian@epa.gov](mailto:gilmour.ian@epa.gov)

**Table S1.** *Hematological parameters of mice exposed to the gun firing smoke-related samples at 4 and 24 h post-exposure.*

|  | WBC  (×10^3^/ul) | RBC  (×10^6^/ul) | HB  (g/dl) | HCT  (%) | MCV  (fl) | MCH  (pg) | MCHC  (g/dl) | RDW  (%) | PLT  (×10^3^/ul) | PCT  (%) | MPV  (fl) | NE  (%) | LY  (%) | MO  (%) | EO  (%) | BA  (%) |
| --- | --- | --- | --- | --- | --- | --- | --- | --- | --- | --- | --- | --- | --- | --- | --- | --- |
| 4 h post-exposure |  |  |  |  |  |  |  |  |  |  |  |  |  |  |  |  |
| Rifle-Single | 2.1±0.4 | 8.8±0.4 | 14.7±0.7 | 52.3±2.7 | 59.3±1.3 | 16.6±0.2 | 28.1±0.5 | 15.1±0.3 | 827±139 | 421±72 | 5.1±0.1 | 18.0±1.9 | 71.6±3.2 | 7.7±1.1 | 2.4±0.8 | 0.3±0.2 |
| Rifle-Burst | 3.2±0.3 | 8.8±0.2 | 14.7±0.5 | 50.3±2.4 | 57.0±1.7 | 16.7±0.2 | 29.4±0.7 | 15.5±0.2 | 745±112 | 380±59 | 5.1±0.1 | 13.5±1.8 | 78.3±2.4 | 5.9±0.6 | 1.8±0.3 | 0.5±0.2 |
| Handgun-Single | 2.4±0.5 | 8.3±0.1 | 13.7±0.3 | 48.8±1.4 | 59.1±1.5 | 16.6±0.3 | 28.2±0.4 | 15.5±0.2 | 805±107 | 408±51 | 5.1±0.1 | 15.5±2.0 | 74.0±2.6 | 7.3±0.2 | 2.3±0.8 | 0.8±0.4 |
| Saline | 2.7±0.8 | 8.1±0.4 | 13.5±0.4 | 46.8±2.1 | 58.0±1.1 | 16.7±0.3 | 28.9±0.6 | 15.5±0.1 | 792±49 | 407±27 | 5.2±01 | 18.9±2.9 | 70.7±4.0 | 7.1±0.8 | 2.6±1.1 | 0.7±0.4 |
| LPS | 4.4±0.5 | 8.4±0.1 | 14.1±0.2 | 49.5±0.7 | 59.0±0.3 | 16.8±0.2 | 28.4±0.3 | 15.2±0.1 | 850±25 | 429±12 | 5.1±0.1 | 19.6±2.2 | 69.0±2.4 | 9.9±0.9 | 1.3±0.4 | 0.2±0.1 |
| 24 h post-exposure |  |  |  |  |  |  |  |  |  |  |  |  |  |  |  |  |
| Rifle-Single | 1.6±0.2 | 8.9±0.2 | 14.5±0.2 | 51.4±0.8 | 57.6±1.2 | 16.2±0.2 | 28.2±0.3 | 15.1±0.2 | 878±55 | 435±21 | 5.0±0.1 | 32.1±8.1 | **56.8±9.5*** | 7.5±2.0 | 3.0±1.3 | 0.7±0.2 |
| Rifle-Burst | 1.6±0.2 | 9.3±0.2 | **15.2±0.2*** | **55.9±0.8*** | 60.4±1.5 | 16.5±0.5 | 27.2±0.4 | 15.3±0.2 | 1067±83 | 560±42 | 5.3±0.0 | 27.6±3.6 | 62.4±2.6 | 8.1±1.1 | 1.6±0.6 | 0.3±0.2 |
| Handgun-Single | 1.9±0.3 | 8.4±0.1 | 13.8±0.2 | 49.6±1.4 | 58.9±1.4 | 16.4±0.1 | 27.9±0.6 | 15.0±0.1 | 940±51 | 468±26 | 5.0±0.1 | 15.8±3.0 | 75.0±3.5 | 6.9±0.5 | 1.9±0.7 | 0.3±0.1 |
| Saline | 1.7±0.2 | 8.6±0.2 | 14.0±0.2 | 49.7±0.9 | 57.7±0.7 | 16.3±0.2 | 28.3±0.2 | 15.4±0.2 | 944±63 | 472±26 | 5.0±0.1 | 16.1±1.8 | 76.5±2.3 | 5.7±1.0 | 1.5±0.5 | 0.2±0.1 |
| LPS | **3.0±0.4*** | 8.6±0.3 | 14.5±0.3 | 52.0±1.8 | 60.7±0.8 | 17.0±0.4 | 27.9±0.2 | 15.2±0.2 | 980±72 | 499±35 | 5.1±0.2 | 22.3±4.2 | 66.4±4.2 | 9.1±1.1 | 1.7±0.4 | 0.5±0.1 |
| 4 h post-exposure |  |  |  |  |  |  |  |  |  |  |  |  |  |  |  |  |
| Rifle-Burst | 3.3±0.5 | 8.1±0.4 | 13.6±0.7 | 45.5±2.7 | 56.0±1.2 | 16.8±0.1 | 30.1±0.5 | 14.9±0.3 | 658±146 | 321±73 | 4.9±0.1 | 20.4±1.8 | 73.1±2.7 | 3.4±1.0 | 2.7±0.7 | 0.4±0.1 |
| Cu Particle | 2.7±0.4 | 8.5±0.2 | 13.8±0.3 | 47.0±1.3 | 55.5±0.9 | 16.3±0.2 | 29.3±0.5 | 15.7±0.3 | 880±58 | 443±26 | 5.1±0.1 | 15.8±2.7 | 77.0±2.9 | 4.8±0.4 | 1.7±0.4 | 0.7±0.3 |
| Saline | 3.3±0.4 | 7.5±0.4 | 12.1±0.7 | 40.0±2.6 | 53.6±1.1 | 16.2±0.5 | 30.3±0.6 | 15.6±0.5 | 667±136 | 343±72 | 5.1±0.1 | 18.1±1.9 | 71.2±3.3 | 5.8±1.1 | 3.7±0.8 | 1.2±0.3 |
| Rifle-Burst +Pen | 2.9±0.4 | 7.5±0.6 | 12.4±0.8 | 41.3±3.2 | 55.3±1.8 | 16.6±0.4 | 30.2±1.1 | 15.6±0.4 | **614±97*** | **297±48*** | 4.8±0.1 | 15.9±3.5 | 74.5±4.5 | 6.1±1.6 | 2.6±0.6 | 1.0±0.3 |
| Cu Particle +Pen | 2.5±0.3 | 8.1±0.2 | 13.3±0.3 | 44.6±1.5 | 54.9±1.7 | 16.3±0.2 | 29.9±0.6 | 15.4±0.4 | 849±96 | 438±37 | 5.3±0.2 | 19.5±1.0 | 72.7±1.7 | 5.0±0.6 | 2.5±0.9 | 0.3±0.1 |
| Saline +Pen | 2.6±0.5 | 8.3±0.1 | 13.6±0.3 | 45.6±1.0 | 54.7±1.1 | 16.3±0.3 | 29.8±0.8 | 15.2±0.2 | 995±50 | 492±31 | 4.9±0.1 | 16.1±1.9 | 76.6±3.5 | 4.1±0.4 | 2.7±1.1 | 0.5±0.2 |
| 24 h post-exposure |  |  |  |  |  |  |  |  |  |  |  |  |  |  |  |  |
| Rifle-Burst | 2.2±0.5 | 8.8±0.3 | 13.7±0.5 | 46.6±1.4 | 53.4±1.5 | 15.6±0.4 | 29.4±0.8 | 15.2±0.2 | 858±42 | 442±18 | 5.2±0.1 | **22.2±1.6*** | **68.9±1.6*** | 6.1±0.8 | 2.1±0.2 | 0.7±0.2 |
| Cu Particle | 2.9±0.3 | 8.3±0.2 | 13.2±0.2 | 44.6±0.9 | 53.9±1.3 | 16.0±0.2 | 29.7±0.8 | 14.9±0.1 | 1017±69 | 492±30 | 4.9±0.0 | **23.5±1.9*** | **70.7±1.9*** | 4.4±0.7 | 1.1±1.1 | 0.2±0.1 |
| Saline | 3.5±0.5 | 8.4±0.1 | 13.4±0.2 | 46.2±1.0 | 55.4±1.8 | 16.0±0.2 | 29.0±0.9 | 15.3±0.2 | 945±47 | 472±27 | 5.0±0.1 | 12.8±2.2 | 80.0±2.8 | 4.3±0.3 | 2.4±0.5 | 0.5±0.1 |
| Rifle-Burst +Pen | 2.6±0.4 | 8.2±0.3 | 13.4±0.7 | 45.6±1.6 | 55.6±1.0 | 16.2±0.6 | 29.3±1.2 | 15.2±0.3 | 723±121 | 348±57 | 4.8±0.1 | **29.7±3.9*** | **55.8±3.4*** | **10.5±1.1*** | 3.1±1.2 | 0.9±0.5 |
| Cu Particle +Pen | 2.3±0.3 | 7.9±0.5 | 12.2±0.8 | 41.4±2.9 | 52.2±1.2 | 15.5±0.4 | 29.7±1.2 | 15.4±0.2 | 764±92 | 372±50 | 4.8±0.1 | 18.0±1.9 | 71.8±2.6 | 5.3±0.9 | 4.0±1.1 | 0.9±0.3 |
| Saline +Pen | 2.9±0.3 | 8.0±0.2 | 12.6±0.5 | 43.1±1.1 | 53.7±0.9 | 15.7±0.4 | 29.3±1.1 | 15.3±0.3 | 697±93 | 352±48 | 5.1±0.1 | 14.8±2.1 | 77.5±2.9 | 4.9±0.7 | 2.2±0.5 | 0.6±0.2 |
| 4 h post-exposure |  |  |  |  |  |  |  |  |  |  |  |  |  |  |  |  |
| Rifle-Burst | 3.2±0.4 | 8.3±0.4 | 13.1±0.5 | 47.9±3.0 | 57.8±1.4 | 15.9±0.5 | 27.6±0.9 | 14.7±0.2 | 775±102 | 373±49 | 4.8±0.1 | 17.6±3.1 | 71.5±3.2 | 6.4±1.1 | 3.2±0.9 | 1.3±0.3 |
| Water-Soluble | 2.9±0.5 | 8.6±0.2 | 13.4±0.2 | 48.1±1.1 | 55.6±0.5 | 15.6±0.3 | 28.0±0.5 | 15.4±0.3 | 832±48 | 406±19 | 4.9±0.1 | 30.3±7.2 | 61.2±6.9 | 5.1±0.7 | 2.7±0.3 | 0.7±0.1 |
| Low-Metal | 3.2±0.8 | 8.5±0.3 | 13.7±0.5 | 48.3±2.1 | 56.8±1.4 | 16.1±0.3 | 28.4±0.4 | 14.9±0.2 | 908±52 | 438±33 | 4.8±0.1 | 21.9±4.2 | 72.5±3.9 | 3.1±0.7 | 2.1±0.9 | 0.4±0.2 |
| Water-Insoluble | 3.3±0.6 | 8.8±0.2 | 14.2±0.3 | 50.6±1.2 | 57.4±0.9 | 16.1±0.2 | 28.1±0.5 | 14.6±0.2 | 810±69 | 394±32 | 4.9±0.1 | 22.4±3.3 | 70.1±3.9 | 3.5±0.3 | 3.1±0.8 | 0.8±0.3 |
| Saline | 3.0±0.4 | 8.2±0.3 | 13.1±0.5 | 45.5±2.3 | 55.4±1.1 | 16.0±0.1 | 28.8±0.5 | 14.9±0.2 | 703±108 | 348±57 | 4.9±0.1 | 19.0±2.1 | 71.8±4.0 | 5.0±1.2 | 3.3±0.9 | 0.8±0.3 |
| 24 h post-exposure |  |  |  |  |  |  |  |  |  |  |  |  |  |  |  |  |
| Rifle-Burst | 2.5±0.6 | 6.9±0.5 | 10.8±0.9 | 39.3±2.9 | 56.6±0.8 | 15.5±0.3 | 27.3±0.6 | 14.4±0.2 | 482±136 | 224±64 | 4.6±0.1 | 20.8±1.3 | 64.6±2.6 | 9.6±0.9 | 3.9±0.7 | 1.1±0.4 |
| Water-Soluble | 2.6±0.4 | 7.8±0.6 | 12.1±0.9 | 43.4±3.2 | 55.4±0.9 | 15.4±0.3 | 27.8±0.8 | 15.4±0.4 | 644±162 | 313±80 | 4.8±0.1 | 24.7±4.6 | 63.4±7.0 | 5.6±0.6 | 5.1±1.9 | 1.3±0.7 |
| Low-Metal | 3.4±0.6 | 8.1±0.3 | 12.7±0.5 | 45.8±1.8 | 56.3±0.5 | 15.8±0.2 | 27.7±0.5 | 14.8±0.1 | 744±88 | 356±43 | 4.8±0.0 | 18.1±3.3 | 68.4±7.4 | 5.1±1.1 | 6.5±2.5 | 1.8±1.3 |
| Water-Insoluble | 2.9±0.7 | 8.4±0.4 | 13.0±0.6 | 46.6±1.7 | 55.8±0.8 | 15.5±0.4 | 27.8±0.6 | 15.0±0.2 | 768±71 | 373±35 | 4.9±0.1 | 25.2±2.4 | 64.8±6.3 | 4.2±0.8 | 4.2±2.7 | 1.6±1.2 |
| Saline | 2.8±0.4 | 8.3±0.5 | 12.5±0.9 | 46.1±3.1 | 55.4±1.2 | 15.0±0.3 | 27.1±0.6 | 14.9±0.2 | 684±148 | 350±76 | 5.0±0.1 | 19.9±1.8 | 70.6±3.2 | 6.1±2.0 | 2.6±0.6 | 0.8±0.3 |

WBC: white blood cell, RBC: red blood cell, HB: hemoglobin, HCT: hematocrit, MCV: mean cell volume, MCH: mean cell hemoglobin, MCHC: mean corpuscular hemoglobin concentration, RDW: red cell distribution width, PLT: platelet, PCT: plateletcrit, MPV: mean platelet volume, NE: neutrophils, LY: lymphocytes, MO: monocytes, EO: eosinophils, BA: basophils, **p*<0.05 compared with saline-exposed group (a negative control) from the same time point. Mice exposed to 2 µg of lipopolysaccharide (LPS) served as a positive control.


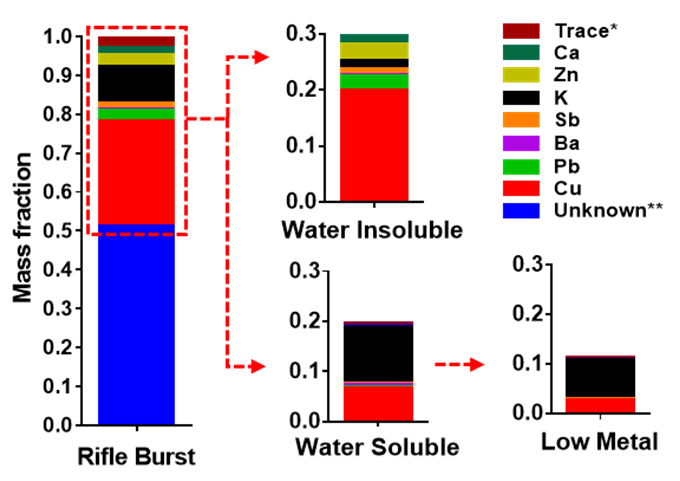


**Figure S1.** *Mass fractions of inorganic elements in water-soluble and -insoluble components of the rifle smoke PM.*  The rifle PM in saline was centrifuged to separate water-soluble components (supernatant) from -insoluble components (pellet). The supernatant was further mixed with Chelex (metal chelator) to remove water-soluble metals from the soluble components (low-metal). *Sum of the rest of quantified elements.**Expected sum of unquantified elements such as N, O, C, and H..
